# What Point-of-Use Water Treatment Products Do Consumers Use? Evidence from a Randomized Controlled Trial among the Urban Poor in Bangladesh

**DOI:** 10.1371/journal.pone.0026132

**Published:** 2011-10-20

**Authors:** Jill Luoto, Nusrat Najnin, Minhaj Mahmud, Jeff Albert, M. Sirajul Islam, Stephen Luby, Leanne Unicomb, David I. Levine

**Affiliations:** 1 RAND Corporation, Santa Monica, California, United States of America; 2 International Centre for Diarrheal Disease Research, Bangladesh (ICDDR,B), Dhaka, Bangladesh; 3 Bangladesh Institute of Development Studies, Dhaka, Bangladesh; 4 Aquaya Institute, San Francisco, California, United States of America; 5 Haas School of Business, University of California, Berkeley, California, United States of America; Hopital Raymond Poincare - Universite Versailles St. Quentin, France

## Abstract

**Background:**

There is evidence that household point-of-use (POU) water treatment products can reduce the enormous burden of water-borne illness. Nevertheless, adoption among the global poor is very low, and little evidence exists on why.

**Methods:**

We gave 600 households in poor communities in Dhaka, Bangladesh randomly-ordered two-month free trials of four water treatment products: dilute liquid chlorine (sodium hypochlorite solution, marketed locally as Water Guard), sodium dichloroisocyanurate tablets (branded as Aquatabs), a combined flocculant-disinfectant powdered mixture (the PUR Purifier of Water), and a silver-coated ceramic siphon filter. Consumers also received education on the dangers of untreated drinking water. We measured which products consumers used with self-reports, observation (for the filter), and chlorine tests (for the other products). We also measured drinking water's contamination with *E. coli* (compared to 200 control households).

**Findings:**

Households reported highest usage of the filter, although no product had even 30% usage. *E. coli* concentrations in stored drinking water were generally lowest when households had Water Guard. Households that self-reported product usage had large reductions in *E. coli* concentrations with any product as compared to controls.

**Conclusion:**

Traditional arguments for the low adoption of POU products focus on affordability, consumers' lack of information about germs and the dangers of unsafe water, and specific products not meshing with a household's preferences. In this study we provided free trials, repeated informational messages explaining the dangers of untreated water, and a variety of product designs. The low usage of all products despite such efforts makes clear that important barriers exist beyond cost, information, and variation among these four product designs. Without a better understanding of the choices and aspirations of the target end-users, household-based water treatment is unlikely to reduce morbidity and mortality substantially in urban Bangladesh and similar populations.

## Introduction

A number of careful studies suggest that treating household drinking water at the point of use (POU) could prevent many of the infant and child deaths attributable to waterborne illness in developing countries [1]–[3]. Nevertheless, household water treatment products such as chlorine or a water filter are very rarely used by the global poor (although boiling is common in a few nations [4]. There is little evidence on what does (or could) induce poor consumers to use POU products on a sustained basis. Thus, our knowledge of factors promoting and impeding adoption of POU products is based on anecdotal reporting of field activities, a “gray” literature of unpublished reports [5]–[9] and a published article that collates the scattered documentation of sustained product use from epidemiological studies [10]–[12]. While each report adds value, there is room to improve our understanding of the preferences for and barriers impeding use of different POU products among poor consumers.

In this research we analyze how often poor consumers in Dhaka, Bangladesh use four POU products and measure their product preferences after they have experience with each product. Along with a companion study [13], this is one of the first attempts to generate rigorous evidence of how urban households use POU products when multiple products are made available.

## Methods

### Ethics Statement

Participants were briefed on the details of the study and afforded opportunity to ask questions and receive answers to those questions. Enumerators obtained informed written consent from each respondent prior to inclusion in the study. This study was reviewed and approved by the Ethical Review Committee at ICDDR,B and the Committee for the Protection of Human Subjects at the University of California, Berkeley.

The sponsors of the study had no role in study design, data collection, data analysis, data interpretation, or writing of the report. All authors had access to all the data in the study. DL and SL had final responsibility for the decision to submit for publication.

### Products

**Figure 1:**
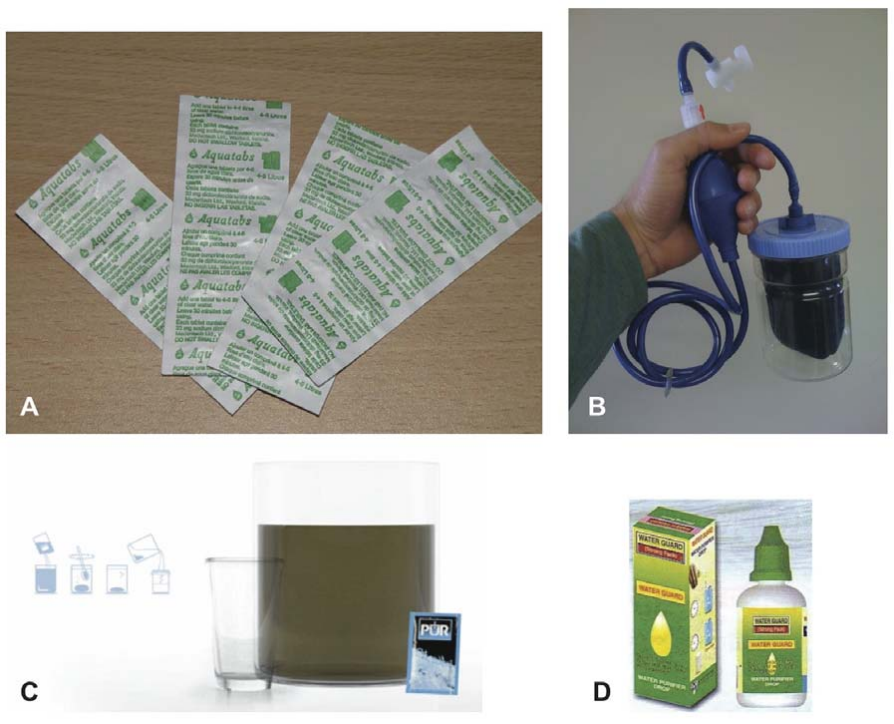
Tested POU Products. Aquatabs (A), the CrystalPur siphon filter (B), the PUR Purifier of Water flocculant/disinfectant mixture (C), and dilute hypochlorite solution branded as Water Guard (D).

This study examines usage of and preferences for four point-of-use water treatment products. Three of the products, which we refer to as the “chemical products,” rely on chlorine for disinfection, including: 1) locally produced and marketed liquid sodium hypochlorite (branded as Water Guard by BioChemical), 2) sodium dichloroisocyanurate tablets (branded as Aquatabs by Medentech, Ltd.), and 3) a combined flocculant-disinfectant powdered mixture (branded as PUR® Purifier of Water by the Procter & Gamble Company). The fourth product was a siphon-driven porous ceramic filter (branded as the CrystalPur Filter by Enterprise Works/VITA) (Figure 1). Each product (or a close variant, in the case of the CrystalPur, for which this is the first field trial) dramatically reduces concentrations of pathogen indicators in drinking water [13]–[16]. The CrystalPur filter is distinct from the more common gravity-driven filters because it utilizes a siphon-driven pressure gradient to draw water through the filter element. (The manufacturer of the product provided third-party laboratory results from Waterlaboratorium Noord (May 2008) indicating >5 log_10_ reduction of *E. coli* in two tested filters. We replicated similar *E. coli* reduction in our own laboratory tests.) Meanwhile, a recent meta-analysis of 31 POU product studies yields a pooled estimate of 42% (95% CI: 33−50%) reduction in diarrheal disease risk [17]. A range of liquid and tablet chlorine products (under various brand names) were available locally at the time of our study.

We recommended each 10 liters of drinking water be treated with 4 drops of Water Guard (a 5.25% concentration), one Aquatab, or one sachet of PUR. Users could add Aquatabs and Water Guard to the container they use to carry water from an outside tap to their home. In contrast, PUR requires a second vessel and a cloth to complete the treatment process. (Our study did not provide storage vessels as a part of the intervention.) The recommended wait-time for treatment using the chemical products is 30 minutes.

The siphon filter can sit in the stored water container if users are willing to wait to draw water through the filter when they want to drink or use the filtered water. Alternatively (and more commonly in our setting) users can filter water from a transport container into a storage container. The filter has a production rate of up to 4 L/hr, declining to 1 L/hr as the water level in the vessel declines and as solids accumulates within the filter. Users have several means maintenance options to restore filter flow after it accumulates solids including cleaning the filter's sleeve, backwashing, and scrubbing the ceramic surface with an abrasive.

The chlorine-based products all provide protection against recontamination until all the free chlorine has reacted with the walls of the storage vessel or with contaminants and metals in the water. If a substantial share of the chlorine reacted with ammonia in the source water, the resulting chloramines still provide some residual protection against recontamination even when the free chlorine is gone.

### Experimental Design

We conducted this research in low-income neighborhoods in the densely-populated mixed-income community of Mirpur within Dhaka (see Figure S1). At baseline we first selected several neighborhoods that survey staff knew to be relatively poor and whose residents frequently present themselves at ICDDR,B's clinic for diarrheal treatment. The field team began at one end of each neighborhood and selected every fifth household. If there was a child under 5 years old, enumerators conducted interviews on basic assets, water supply, water treatment, sanitation, and hygiene behaviors, and if not, they approached the next closest household. The female head of household was interviewed in 98% of cases because women in this setting typically have responsibility for water treatment. The baseline sample consisted of 800 households.

After completing the baseline survey, enumerators explained the health dangers posed by untreated local water. For example, enumerators explained, “Human feces can enter the water as a result of faulty pipes introducing contamination from the environment. This means that even before the water gets to your household, it can be contaminated. Also, water can become contaminated easily within the home, for instance by not keeping your drinking water storage containers clean and covered at all times or by dipping your hands into the container to draw water.” Enumerators then provided detailed presentations of the four POU products in randomized order and asked households to rank their preferences and state their willingness-to-pay for each product.

After the baseline survey, 200 of the 800 households were randomly selected as controls. Their participation in the baseline ended at this point. For the 600 treatment households, enumerators then provided one of the four products for a two-month free trial. The order of the product trials was randomized.

During the two-month product trials a separate team of technicians visited both treatment and control households to collect stored drinking water samples and ask a few questions about water collection and treatment behaviors. These visits took place roughly one to four weeks after the baseline survey and introduction of the first product and 4 to 8 weeks after later survey rounds and product introductions.

At the end of each two-month trial period, enumerators visited each treatment household for a follow-up survey to measure self-reported product usage and updated product preferences as well as answer questions about their experience with the product. Each household was then assigned a new product in random order. The cycle was repeated four times, so that over 8 months every treatment household had a two-month trial with each of the 4 products in random order.

Enumerators visited both treatment and control households at the final survey round to collect information on final product preferences. A companion article [18] compares the preferences and willingness-to-pay of controls with interventions households in order to examine how hands-on experience affects consumer valuations for these health products.

### Water Quality Analysis

We analyzed multiple measures of product usage. Most directly, we asked users to self-report product usage both at the water collection visit and at the survey. Because courtesy bias can lead to over-reported product usage [19], we also analyzed several objective indicators of product usage.

We measured the concentration of *E. coli* in water stored at the household. At the water collection visit we collected stored water samples in autoclaved bottles and used cold boxes to transport the samples to the laboratory at ICDDR,B. We assessed the concentration of *E. coli* using the membrane filtration technique [20]. In brief, an aliquot of 100 ml of water was filtered through 45-micron Millipore membrane filters. Filter papers were then placed on modified membrane-thermotolerant *E. coli* agar media and incubated at 35°C for two hours and then at 44.5°C for another 22 hours. Red or magenta colonies were counted.

We utilize three measures of *E. coli* to examine usage and efficacy: fractions of homes with *E.coli* concentrations less than one colony forming unit (CFU) per 100 mL (the WHO-recommended maximum for drinking water, which we also refer to as “no detectable *E. coli*”); fractions of homes with *E. coli* concentrations <10 CFU/100 ml; and the distribution of *E. coli* concentrations (CFU/100 ml). Note that low *E. coli* concentrations (relative to controls) depends on both homeowners using the product and the microbiological effectiveness of the product.

Also, if the user self-reported use of a chemical product (Water Guard, Aquatabs or PUR) during the water collection visit, we tested for residual free chlorine using a color wheel colorimeter (HACH LANGE GmbH, USA). However, even if a household uses one of the chemical products we would not detect free residual chlorine if all the free chlorine had reacted with the storage container or contaminants in the water.

### Sample Size, Enrollment, and Attrition

To detect differences in proportions of product usage of 10 percentage points with 80% power at 95% confidence required a sample size of approximately 100 treatment households per product-trial, for a total of 400 households. We sampled 150 treatment households per product-trial to account for any potential attrition. We also sampled 200 households in the control group.

The study began in January 2009 with 800 participating households and was completed in December 2009 with 755 participating households, resulting in 94% retention, with similar proportions for treatments (95%) and controls (94%). We also collected water quality data but no exit survey for 7 treatment households (1.2%) and 5 control households (2.5%). The most common reason for a household to drop out of the study was outmigration from the community. Attrition does not appear related to a household's first assigned products or other randomized treatment assignments. When we ran a probit regression predicting dropout as a function of all treatment assignments, the joint Chi-squared test was not statistically significant (p-value = 0.24).

Randomization appeared successful. The chi-squared test p-value was 0.67 in a probit regression that predicts treatment versus control as a function of baseline literacy, household size, native Urdu speaker, type of source water, and respondent age and gender. Results on the regressions predicting dropout and randomization are in Appendix S1 (Tables S1 and S2 respectively).

### Data Analysis

Household survey results were recorded in hardcopy forms and double-entered into digital forms using Epi Info (Microsoft Corp., Redmond, WA). Digital data tables were then exported into Stata (StataCorp LP, College Station, TX). Laboratory results were recorded in hard copy and double entered.

All reported confidence intervals, regressions and statistical tests take into account the repeated nature of the sampling by using the sandwich estimator for standard errors using the “cluster” option in Stata.

We often report tests of statistical significance for outcomes at households with one or two of the products versus those households when they had the other products. As there are multiple comparisons possible with four different products, the p-value of a single reported test can have inflated power. To reduce accidental data mining, we do not report comparisons between individual products if results across the four products are not jointly statistically significant.

### The Setting

Only one third of respondents had completed primary school and the majority of per capita household incomes were less than the global poverty line of $2 (in purchasing power parity) per day (data not shown).

The study area is a crowded urban community, with almost all households sharing walls. Most residences have cement floors (82%), cement or tin walls (81%), and a corrugated iron roof (92%).

A substantial minority (45%) of our sample are Urdu-speaking Bihari. The Bihari are Muslims who left Bihar and nearby north Indian states for East Bengal (later East Pakistan) at the partition of British India. In part because most opposed the independence of Bangladesh from Pakistan and many await repatriation to Pakistan, most remain living in refugee-oriented neighborhoods.

At the baseline survey, 73% of treatment households and 76% of controls reported piped water as their main drinking water source (p-value = 0.52). Most of the others store piped water in a cistern for a household or group of houses.

Almost all water stored in the control households was contaminated with *E. coli*. Over all waves, 83% of water samples from control households had detectable *E. coli*, with 33% less than 10 CFU/100 ml (N = 720 observations on 200 households). The mean and median *E. coli* concentrations were 182 and 43.5 CFU/100 ml, respectively.

No controls reported treating their current drinking water with any of the point-of-use products we tested. At the same time, at baseline, 43% of all respondents claimed they treated their drinking water (at least sometimes), with 78% of those mentioned boiling and 41% mentioning filtering through a cloth (multiple responses were allowed). Fewer than 2% of all respondents at baseline mentioned a POU product such as a filter or chlorine.

## Results

### Usage and performance indicators

Table 1 presents several measures of usage and performance for all products averaged over all survey waves. At the water collection visits, we defined self-reported users as those households that report having treated their water in the past 24 hours. For the survey (typically about two weeks after the water collection visit), we defined self-reported users as those who report some or all of their current stored drinking water is treated and that they used their POU product since yesterday.

**Table 1:** Indicators of POU product usage for all products.

		(1)	(2)	(3)	(4)	(5)	(6)	(7)
		Self-Report	Self-Report	Positive Chlorine Test	“No detectable” *E. coli*	*E. coli *<10 CFU/100 ml	Mean *E. coli* CFU/100 mL	Median *E. coli* CFU/100 mL
	Source of data	Survey	Water collection visit	Water Collection Visit	Water Collection Visit	Water Collection Visit	Water Collection Visit	Water Collection Visit
**Aquatabs**	Mean	13%	20%	10%	28%	42%	151	18
	S.E.	(1.4)	(1.8)	(1.2)	(1.9)	(2.2)	(13)	
**Water Guard**	Mean	19%	24%	11%	31%	47%	139	13
	S.E.	(1.6)	(1.9)	(1.3)	(2.0)	(2.2)	(12)	
**PUR**	Mean	7%	10%	3%	24%	41%	159	25
	S.E.	(1.0)	(1.3)	(0.7)	(1.9)	(2.1)	(13)	
**Filter**	Mean	21%	29%	–	24%	43%	163	22
	S.E.	(1.7)	(2.0)		(1.9)	(2.2)	(14)	
**All Products**	Mean	15%	21%	8%	27%	43%	154	20
	S.E.	(0.88)	(1.1)	(0.7)	(1.2)	(1.4)	(8)	
	N	2339	2151	1737	2120	2120	2120	2120
**Controls**	Mean	–	0	0	17%	33%	182	43.5
	S.E.				(1.8)	(2.4)	(14)	
	N		722	720	720	720	720	720

N is the number of household visits for the 600 treatment and 200 control households across four household visits (not including the baseline). Free chlorine was measured only among self-reported users of chemical products, but N in that column refers to number of households with chemical products at that survey round. Self-reports at surveys in column 1 defined as households that report “At least some water treated” and “last used product” is “today” or “yesterday.” Self-reports at water collection visits in column 2 defined as households that report they “Treat drinking water with [POU Product]” and “how long ago did you treat?” ≤24 hours.

The proportion of households with either measure of self-reported usage is somewhat higher than our objective measures of product usage, yet all suggest improvements in water quality relative to controls (Table 1). Across all products, 27% of treatment households exhibited no detectable *E. coli* in stored drinking water, as compared to 17% of controls (P<0.01). The mean and median *E. coli* concentrations in stored drinking water among treatment households (154 CFU/100 mL; 95%CI 138−169, and 20 CFU/100 mL) were also lower than that of control households (182 CFU/100 mL; 95%CI 153−210; P = 0.09 on test of means; 43.5 CFU/100 mL, P<0.001 on a Wilcoxon-Mann-Whitney test of medians).

### Self-reported usage

Combining all study waves, households at the water collection visit were most likely to self-report using the filter (29%, 95% CI: 25−32%). The share reporting using Water Guard (24%, 95% CI: 20−27%) and Aquatabs (20%, 95% CI: 17−24%) were similar (and statistically indistinguishable; P = .13) from each other, but were both statistically significantly lower than for the filter (P<.01). PUR had the lowest share of self-reported usage at the water collection visit (10%, 95% CI: 8−13%, difference significant at P<0.01 on four-way adjusted Wald test).

### Chlorine tests

Among households assigned a chemical, those with Water Guard had a similar proportion of positive chlorine tests (11%) as households assigned Aquatabs (10%, P = 0.58). Both proportions were statistically significantly higher than for PUR (3%, P<0.001).

### Microbiological performance among all households receiving a product

Among our three objective measures of product performance and usage, only the share of households with no detectable *E. coli* was significantly different across all 4 products and thus allowed product-by-product comparisons. Of households assigned Water Guard, 31% had no detectable *E. coli* and the median *E. coli* concentration was 13 CFU/100 mL. Households assigned Aquatabs had no detectable *E. coli* 28% of the time (difference with Water Guard not significant, P-value = 0.18) and a median *E. coli* count of 18 CFU/100 mL. When households were assigned either Water Guard or Aquatabs they exhibited less microbiological contamination than when the same households were assigned PUR (24% no detectable *E. coli*; P = 0.03 on three-way test across chemicals for no detectable *E. coli*; median *E. coli* of 25 CFU/100 mL).

The story is more complex for the filter, which had slightly higher self-reported usage at the water collection visit (29%) than any other product. In contrast, only 24% of households assigned the filter had no detectable *E. coli*, which was statistically significantly lower than for Water Guard (31%, P<0.05), marginally lower than the share for households assigned Aquatabs (28%, P = 0.14), and about the same as the share for households assigned PUR (24%, P = 0.90). The median *E. coli* concentration at filter household visits was 22 CFU/100 mL.

### Microbiological performance among self-reported users

**Figure 2:**
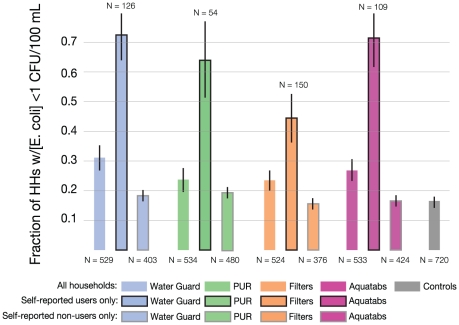
Percent of households with stored water samples with no detectable *E. coli*, by assigned product and by self-reported usage in last 24 hours at water collection visits. Error bars correspond to standard error of mean.

The previous section analyzed microbiological outcomes for all households *assigned* a product, and therefore includes non-users of each product. The products appear substantially more effective when we focus on the non-random subset of households that reported they *used* each product (Figure 2).

On average, the *E. coli* contamination of those at the water collection visit who reported using the POU product in the past 24 hours is far below that of non-users or of controls. For example, the median self-reported user had no detectable *E. coli* in their stored water, which is far below the median of 34 CFU/100 mL for self-reported non-users and 43.5 CFU/100 mL for control households (P<0.01).

Among the households self-reporting usage of the chemical products, about 70% had no detectable *E. coli*, which was a higher share than the 45% of filter self-reported users without detectable *E. coli* (difference P<0.01, see Fig. 2). A much lower 17% of controls had no detectable *E. coli*, which was similar to the share among self-reported non-users of each product (15–20%). The higher rates of detectable *E. coli* for self-reported non-users and for controls relative to self-reported users were statistically significant for all products. Differences between controls and non-users and among non-users of different products were not statistically significant.

Self-reported product usage resulted in a roughly 1.3 log_10_ reduction in *E. coli* concentration in stored water as compared to controls: average log_10_ E. coli was roughly 1.6 points lower for Water Guard, 1.4 points lower for Aquatabs, 1.2 points lower for PUR, and 0.9 points lower for the filter (with differences compared to controls all significant at the 1% level; we assign a log_10_ value of -1 to those observations with no detectable *E. coli* to retain them in the analysis). These water quality improvements are very similar when comparing self-reported users to controls or to self-reported non-users, respectively.

Self-reported users of chemical products often (57% of the time) had no detectable chlorine, but even without detectable chlorine they were nearly three times more likely than controls to have no detectable *E. coli* in stored water (47% vs. 17%, respectively, with similar proportions across the three chemical POU products). Of the 43% of self-reported users of the chemical products for whom we detected chlorine residual, none of the 124 samples exhibited detectable *E. coli*. The comparisons between users and non-users (among the treatment households) and between users and controls can be biased estimates of causal effects of product use if there is self-selection of who uses these products. For example, if users have less safe water, the causal effects will be larger than those seen in the comparisons in Figure 2. In fact, the almost-identical mean and median *E. coli* concentrations for controls and for intervention households who report they did not use a POU product during a free trial suggests those with more (or less) contaminated source water are not more likely to use a POU product. As an additional check, we use intervention status as an instrumental variable to estimate the effect of being assigned a safe-water POU product on the intervention group [21]. Results were very similar to those shown in Figure 2 (Table S3).

### Survey evidence on barriers to usage of POU products

In Table 2 and Table 3 we provide nonexperimental results from survey questions to explore reasons why treated households may choose not to use a POU product during the free trial. Table 2 considers usage rates separately for households with greater or less baseline concern for diarrhea as a cause of illness, and greater or less baseline awareness about the causes of diarrhea. Households are labeled as having greater baseline concern for diarrhea if they freely named diarrhea as the most pressing disease affecting their neighborhood; households are labeled as “highly aware” if they correctly named four or more ways to avoid diarrhea, and “low awareness” households could name at most two ways of avoidance at baseline prior to our informational script. Table 3 presents descriptive evidence on the most commonly cited barriers to POU product usage for all follow-up survey waves combined. It presents separately by product the rates at the follow-up visits that households freely named taste or smell of the treated water and wait time when asked for the biggest obstacles to water treatment (no other barriers were cited by more than 5% of household visits).

**Table 2:** Usage Rates Across All POU Products, Split by Baseline Predictors of Usage.

		(1)	(2)	(3)	(4)
		Share of Treated Households (Number of Observations)	Positive Chlorine Test	“No detectable” E. coli	Self Reports POU usage at water collection visits
Baseline high level of concern	Yes	51.5% (291)	9%	27%	22%
	No	48.5% (309)	7%	26%	19%
	Wald test p-value		.222	.914	.256
Baseline high level of awareness	Yes	23% (138)	8%	28%	21%
	No	45% (271)	7%	25%	20%
	Wald test p-value		.846	.322	.706

Number of observations in column 1 varies by outcome considered. For the outcome “baseline high level of concern” it is the number of households among the 600 treatment households at baseline, while for “baseline high level of awareness” the 191 treated households that named exactly 3 correct ways to avoid diarrhea at baseline are omitted. Free chlorine was measured only among self-reported users of chemical products. Self-reports at water collection visits in column 4 defined as households that report they “Treat drinking water with [POU Product]” and “how long ago did you treat?” ≤24 hours.

**Table 3:** Nonexperimental Survey Evidence on Barriers to Usage of POU Products.

	(1)	(2)
	Dislike Taste/Smell	Too Much Time
Aquatabs	50%	1%
Water Guard	56%	2%
Pur	57%	20%
Filter	6%	27%
Average over all products	43%	12%

Rates at which treated households named either a dislike for the taste/smell of the treated water of a product (column 1) or the necessary wait time for safe water (column 2) at all follow-up rounds. The precise survey question was, “What were the biggest obstacles to use the [PRODUCT] every time water was collected?” Respondents were asked as an open response and results recorded and categorized. No other complaints were named by more than 5% of respondents.

## Discussion

Our main results are as follows:

- Even with four bimonthly household visits explaining the health hazards of untreated drinking water, and free trial periods, even the most popular product (the filter) exhibited less than 30% usage.
- The siphon filter was generally self-reported to be used slightly more than Water Guard and Aquatabs, and all were used substantially more than PUR.
- All products were very effective at reducing *E. coli* concentrations when used, although self-reported users of the filter had somewhat higher rates of detectable *E. coli* than self-reported users of the chemical products.

There are several possible explanations for the 53% of self-reported users of chemicals with no detectable free chlorine but detectable *E. coli*: imperfect recall or a courtesy bias leading to over-stated recent product use, incorrect product usage, or consumption of the free chlorine followed by water handling that leads to recontamination [22], [23]. Yet because the 57% of self-reported users of the chemical products for whom we could not detect chlorine were nearly three times as likely as controls to produce no detectable *E. coli* in stored water (47% vs. 17%), it suggests that many self-reported users of chemicals are truthfully reporting product use. (Comparisons of *E. coli* between product users and non-users can be biased measures of product effectiveness if usage depends on *E. coli* contamination or factors correlated with contamination. In our data self-reported non-users have rates of no detectable *E. coli* between 15 and 20%, all very close to the 17% of controls with no detectable *E. coli*. Thus, self-selection does not appear to be important.)

Recontamination may also explain some of the filter users with detectable *E. coli* in their stored water. In our laboratory results the filter was effective at eliminating all detectable *E. coli*, yet in the field over half of the samples of stored water from self-reported users of the filter had detectable *E. coli* (as compared to about 30% of self-reported chemical users having detectable *E. coli*, and none of the chemical users whose stored water contained measurable chlorine residual). Users of the chemical products may have had more success in maintaining water without detectable *E. coli* because chlorine residual (and perhaps by-products of chlorine reactions such as chloramines) minimizes recontamination after treatment.

These results reinforce the familiar advice that safe storage is an important complement to point-of-use water treatment, particularly for POU products such as water filters that provide no lasting protection.

While the above discussion emphasizes comparisons across products, our most striking result is the low usage of even the most popular products. Most theories of health decision-making identify consumers' lack of information on the risks of untreated water coupled with product cost as key constraints on household water treatment [24]–[26]. Our intervention addressed these barriers: Price was zero during the free product trials and there were multiple household visits providing information that untreated water is dangerous and these products can effectively reduce that danger. Nonexperimental evidence from surveys suggests that our informational messages had some effect: the share of treated households reporting that their source water is safe to drink without treatment decreased from 31% at baseline to 21% at exit (P<.001).

Nonetheless, usage of the products was low even among those we might suspect to be more likely to adopt. Households that at baseline expressed greater concern for diarrhea (the 51% that freely named diarrhea as the most pressing health concern in their area) were not more likely to use the products during the study (Table 2); nor did those households with greater baseline awareness about the causes of diarrhea use the products at higher rates (Table 2). And although anecdotally consumers do not like the taste and smell of chlorinated water, taste or smell was named as an obstacle to water treatment just 43% of the time (55% of the time when households had chemical products), while usage of these products was not anywhere near 57% and it is not clear if taste and smell were named as true obstacles discouraging use, or as an ex post justification a household can use when we asked them to explain their lack of product adoption (Table 3). Finally, intervention households self-reported having experimented with the products at least once during free trials nearly 90% of the time, yet just 15% self-reported product usage by the time of the two-month follow-up survey visits. In sum, the persistent low sustained rates of usage of these products makes clear that important barriers exist beyond cost, information, and variation among these four product designs.

Unless demand increases considerably, household water treatment is unlikely to reduce morbidity and mortality substantially in urban Bangladesh. Product design that lowers the cost and promotes the habit of water treatment is likely to be important [27]. Those designing and distributing safe water products must better understand the preferences, choices, and aspirations of the at-risk populations.

Effective marketing will need to go beyond standard messages about water and health (such as those we used). Marketing messages that engage community pride, associate untreated drinking water with ingestion of human feces, build on norms that make consumers ashamed to be seen engaging in unsanitary activities, and build on religious injunctions related to purity should be evaluated to see if they improve uptake.

## Supporting Information

Figure S1 **Study site location, Mirpur, Dhaka, Bangladesh.**
(DOC)

Table S1 **Predicting dropout as a function of treatment assignments.** Probit regression with dependent variable = 1 if a baseline household dropped out before end of study. Standard errors in parentheses.
(DOC)

Table S2 **Test of randomization by predicting treatment status as a function of baseline characteristics.** Dependent variable = 1 if household is assigned to intervention (vs. control) group at baseline. Standard errors in parentheses.
(DOC)

Table S3 **Instrumental variables (IV) regressions for endogenous self-reports on usage.** Column 1 contains first stage results for both IV regressions. Columns 2−3 contain results for “no detectable” *E. coli* (*E. coli* <1 CFU/100 mL) and compares OLS results in column 2 with IV results in column 3. Columns 4−5 make similar comparison for Log_10_(*E. coli*) outcome. Standard errors in parentheses.
(DOC)

Appendix S1 (DOC)
