# Supplementary figures and images for: What Point-of-Use Water Treatment Products Do Consumers Use? Evidence from a Randomized Controlled Trial among the Urban Poor in Bangladesh
Source: PLoS One. 2011 Oct 20;6(10):e26132. doi: 10.1371/journal.pone.0026132 (PMC3197608; doi:10.1371/journal.pone.0026132)

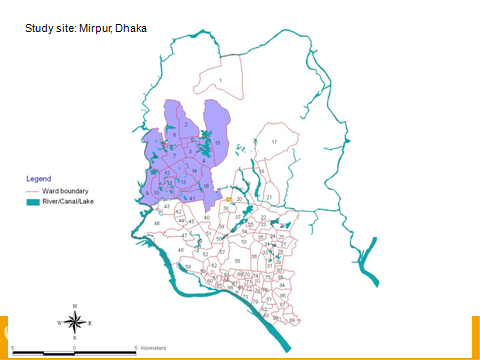

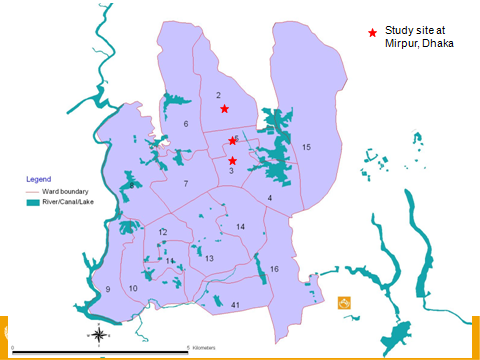

Supplement: Figure S1 — Study site location, Mirpur, Dhaka, Bangladesh. (DOC) [file pone.0026132.s001.doc]

**Table S1**


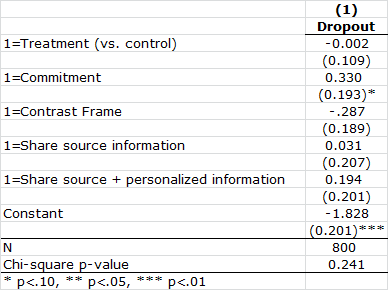

Supplement: Table S1 — Predicting dropout as a function of treatment assignments. Probit regression with dependent variable = 1 if a baseline household dropped out before end of study. Standard errors in parentheses. (DOC) [file pone.0026132.s002.doc]

**Table S2**


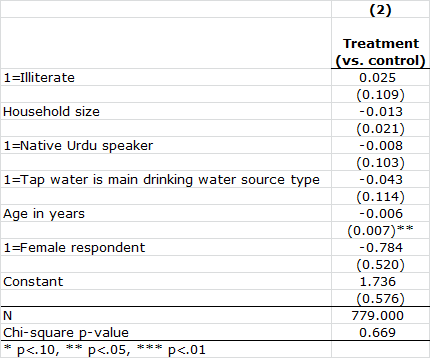

Supplement: Table S2 — Test of randomization by predicting treatment status as a function of baseline characteristics. Dependent variable = 1 if household is assigned to intervention (vs. control) group at baseline. Standard errors in parentheses. (DOC) [file pone.0026132.s003.doc]

**Table S3**


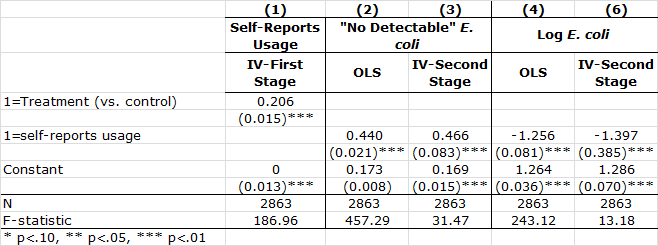

Supplement: Table S3 — Instrumental variables (IV) regressions for endogenous self-reports on usage. Column 1 contains first stage results for both IV regressions. Columns 2−3 contain results for “no detectable” E. coli (E. coli <1 CFU/100 mL) and compares OLS results in column 2 with IV results in column 3. Columns 4−5 make similar comparison for Log10(E. coli) outcome. Standard errors in parentheses. (DOC) [file pone.0026132.s004.doc]
