# Supplementary material for: What Point-of-Use Water Treatment Products Do Consumers Use? Evidence from a Randomized Controlled Trial among the Urban Poor in Bangladesh
Source: PLoS One. 2011 Oct 20;6(10):e26132. doi: 10.1371/journal.pone.0026132 (PMC3197608; doi:10.1371/journal.pone.0026132)
Supplement: Appendix S1 — (DOC) [file pone.0026132.s005.doc]

Figure A1: Study site location.


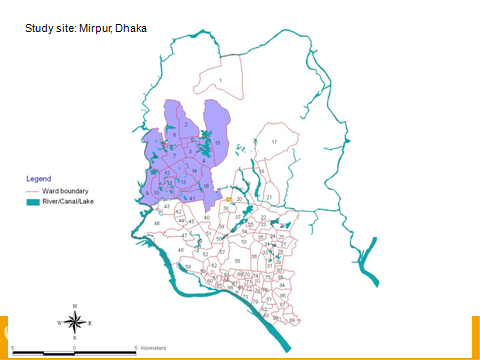

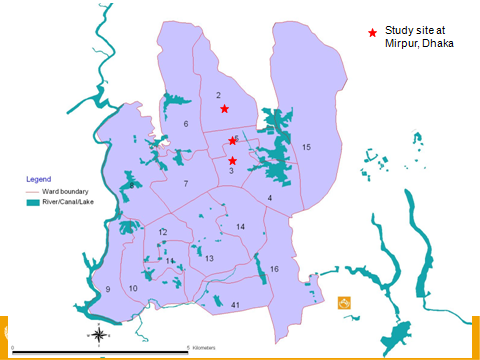


Translated Copy of Auction Experiment: Wilingness to pay for POU products

Today, we are giving you an opportunity to participate in an auction whereby you could buy one of the water purifying products we intrduced to you earlier. Same opportunity will be given to those househods in this area who are taking part in our survey

Have you ever heard about AUCTION?[FRA: Wait for response; then explain clearly the next paragraph]

In an auction, typically, many people participate to buy a certain product and indicate the most they are willing to pay to get the product. In most cases the person who indicates the highest amount of money to buy the product wins and pays that exact sum of money to obtain the product.

Today, by participating in a similar auction you would indicate the most you are willing to pay for each of the water purifying product. We are conducting auction for each of the water purifying products to know the most you are willing to pay for each product. Actually, the product that is to be auctioned today in your case is pre-decided by a lottery as you will be given the opportunity to buy only one product today. The price of that particular product is also drawn from a range of prices by lottery and the price chosen is within the buying capacity of people in this area.

The name of the auctioned product and its pre-decided price is written in a piece of paper and put in a sealed envelope [FRA shows the envelope] and t we would not know it before opening the envelope. This means that the auctioned product and its price can be different for different households in this area.

**For your information, the range of prices from which the price of each product is drawn is as follows:**

**FILTER**: The price of a filter is drawn form a range of prices between 0 Taka to 500 Taka. This means, for example, the drawn price can be any price between 0 to 500; it can either be 0 taka , 50 taka , 100 taka , 110 taka, 120 taka, 150 taka, 170 taka, 200 Taka, 250 Taka, 300 Taka, 400 Taka, or 500 Taka but not more than 500 Taka. If you regularly use a filter like this to treat your household’s drinking water, it will last on average for one year.

**AQUATABS:** The price of a sleeve of Aquatabs is drawn form a range of prices between 0 Taka to 30 Taka. This means, for example, the drawn price can be any price between 0 to 30 Taka; it can either be 0 Taka , 2 Taka, 5 Taka, 10 Taka , 15 Taka , or 25 Taka but not more than 30 Taka. If you regularly use Aquatabs to treat your household’s drinking water, one sleeve of Aquatabs will last on average for two weeks.

**WATERGUARDS:** The price of a bottle of Waterguard is drawn form a range of prices between 0 Taka to 25 Taka. This means, for example, the drawn price can be any price between 0 to 25 Taka; it can either be 0 Taka , 1 Taka , 5 Taka , 10 Taka,or 15 Taka but not more than 25 Taka. If you regularly use Waterguard to treat your household’s drinking water, one bottle will last on average for two weeks.

**PuR**: The price of 5 sachets of PuR is drawn form a range of prices between 0 Taka to 25Taka. This means, for example, the drawn price can be any price between 0 to 25 Taka; it can either be 0 Taka , 5 Taka , 7 Taka , 10 Taka 15 Taka or 25 Taka but not more than 25 Taka. If you regularly use Aquatabs to treat your household’s drinking water, 5 sachets of PuR will last on average for one week.

NOW I WILL EXPLAIN TO YOU HOW I WILL CONDUCT THE AUCTION TODAY

First, I will give you a piece of paper where the name of each product will be written. You will then write next to each product the maximum amount of money that you would be willing to pay to buy each product If you do not like to buy a particular product you could write (indicate) zero price for that product. I will help you to write down the price(s) you indicate, if you are not able to write.

Once you return the paper with your preferred prices written on it for each product, I will open the sealed envelope to know about the product to be auctioned in your case and its lottery-decided price. Then I will compare the price that you wanted to pay for the auctioned product with its lottery-decided price.

If the price that you wanted to pay at most for the auctioned product is greater than its lottery-decided price, then you will WIN the auction and as a result you will buy the product today in cash at its lottery-decided price. Thus, you could buy the auctioned product at a price lower than what you actually wanted to pay at most for that product.

I will now explain you more detail about the auction producedure.

**Do you have any question?[ FRA; answer any question the respondent might have and make everything clear]**

In the auction today, for you the best approach is to mention what you really wanted to pay at most for a product. Suppose, a product is worth to you 10 Taka, then it is unlikely that you would want to pay 15 Taka for it but it is very likely that you would want to pay 8 Taka for it. Now if it turns out that the price of that product in the auction/lottery is 15 Taka, then you would not buy it. However, if it turns out to be 8 Taka, then you would buy it.

If you mention a higher price than what the product is worth to you, then it is likely that you will end up buying the product at a higher price than you actually wanted to pay for the product. On the other hand, if you mention a lower price than the product is worth to you, you will miss the opportunity to buy the product at a price you really wanted to buy the product.

Take for example, the case of a filter. If you mention a price that is higher than what is a filter worth to you, then if you win the auction, you will buy the filter at a higher price than what you actually wanted to pay for the filter. On the other hand, if you offer a lower price than what you actually wanted to pay for the filter, then if you do not win, you will miss an opportunity to buy the filter at your desired price.

**Do you have any question?[ FRA; answer any question the respondent might have and make everything clear]** **If you do not understand anything I said or if you have any question, please let me know, I will explain to you again.**

**FRA: I will conduct the auction in the following way:**

1. I will first give you a piece of paper on which the name of each product is written and numbered as 1 o 4.
2. You will then write next to each product name, the highest amount of Taka you want to pay for that product. [Illiterate respondents will get help from the FRA to write their bid]. If you do not like to buy any particular product, you can always mention zero price for it.
3. You will then return the paper to me.
4. Once you return the paper after writing you preferred prices, I will then open the sealed envelope to know which product is to be auctioned for you today and also know its pre-determined price.
5. Then I will compare the price you mentioned for the auctioned product with its pre-determined price.
6. If the price you mentioned is higher than the predetermined price for the auctioned product, then you will WIN the auction. BUT if the price you mentioned is equal or less than the pre-determined price for the product, you will not WIN the auction.
7. As a winner, however, you will get the auctioned product by paying (in cash) its pre-determined price. This means, if you WIN, you will get the auctioned product at a price lower that what you actually wanted to pay for it.

Q801. Do you have any question before I start the auction procedure?

1. Yes [FRA answer all questions]

2. No

[FRA starts the procedure, once convinced that the respondent understood everything and that she was willing to participate]

FRA now follows the following procedure:

FRA: NOW GIVE THE REPSONDENT THE PIECE OF PAPER CONTAINING THE NAME OF EACH PRODUCT NUMBERED AS 1 TO 4

FRA: GET THE PAPER BACK FROM THE RESPONDENT, ONCE THE RESPONDENT IS DONE WRITING THE WILLINGNESS TO PAY AMOUNT FOR EACH PRODUCT.

FRA: NOW OPEN THE SEALED ENVELOPE TOGETHER WITH THE RESPONDENT AND SHOW THE NAME OF THE AUCTIONED PRODUCT AND DISCUSS ITS PREDETERMINED PRICE.

FRA: NOW LET THE RESPONDENT KNOW THE RESULT OF THE AUCTION

FRA: IF THE RESPONDENT WINS, THEN GIVE HER THE AUCTIONED PRODUCT IN EXCHANGE OF CASH EQUIVALENT TO ITS PRE-DETERMINED PRICE. THEN PUT THE MONEY IN THE ENVELOPE AND SEAL IT IN FRONT OF THE RESPONDENT

FRA: NOW THANK THE RESPONDENT FOR PARTICIPATION AND WRITE DOWN THE ANSWER TO THE FOLLOWING QUESTIONS

Q802a. Product 1 Name---------------

Q802b. Respondents WTP for Product1 ----------------

Q803a. Product 2 Name---------------

Q803b. Respondents WTP for Product 2 ----------------

Q804a. Product 3 Name---------------

Q804b. Respondents WTP for Product 3 ----------------

Q805a. Product 4 Name---------------

Q805b. Respondents WTP for Product4 ----------------

Q806. Name of the auctioned PRODUCT:

1. Aquatabs
2. Filter
3. Waterguard
4. PuR

Q807. Price (predetermined) of the auctioned product----------------------

Q808. Has the respondent won the auction?

1. Yes

2. No

Q809. Did the respondent buy the auctioned product?

1. Yes [Skip to the question Q811]

2. No

3. Received at free of cost

Q810. Why didn’t the respondent buy the auctioned product?

1. The respondent did not have the required amount of money at home

2. The respondent could not take the decision to purchase on her own

3. It was not her preferred product.

4. Received at free of cost

95. Other (Please specify)…………………

Q811. How many persons were present during the auction interview?

a) No of Adult ………

b) No of Child 12 years or below

FRA: WRITE DOWN YOUR OBSERVATIONS ON THE FOLLOWING QUESTIONS

Q812. Has the respondent, who did not win the auction, shown interest to buy the auctioned product at the auctioned price?

1. Yes
2. No
3. Not applicable

Q813. Has the respondent shown interest to buy her preferred product at the price she indicated in the auction, in case the auctioned product was not her preferred one?

1. Yes
2. No
3. Not applicable

FRA : PLEASE WRITE BELOW ANY FURTHER OBSERVATIONS REGARDING THE AUCTION

Predicting Dropout as function of treatment assignments:

| Probit regression |  |  | Number of obs = | 800 |
| --- | --- | --- | --- | --- |
| Log likelihood = -134.1471 | |  | LR chi2(5) = | 6.74 |
|  |  |  | Prob > chi2 = | 0.2407 |
|  |  |  | Pseudo R2 = | 0.0245 |
|  |  | Std. Err. | z | P>z |
| Treatment? (vs. Control) | -0.002 | 0.247 | -0.01 | 0.992 |
| 1=Commitment | 0.330 | 0.193 | 1.71 | 0.087 |
| 1=Contrast Frame | -0.287 | 0.189 | -1.52 | 0.128 |
| 1=Share Source Info | 0.031 | 0.207 | 0.15 | 0.879 |
| 1=Share Source + Own Info | 0.194 | 0.198 | 0.98 | 0.327 |
| Constant | -1.828 | 0.201 | -9.12 | 0.000 |
| Dependent Var: Dropout (=1 if Baseline HH failed to finish study) | | | |  |

Predicting Treatment (vs. Control) at Baseline:

| Probit regression |  |  | Number of obs = | 779 |
| --- | --- | --- | --- | --- |
|  |  |  | LR chi2(6) = | 4.06 |
|  |  |  | Prob > chi2 = | 0.6687 |
| Log likelihood = -428.4569 | |  | Pseudo R2 = | 0.0047 |
|  | Coefficient | Std. Err. | z | P>z |
| 1=Illiterate | 0.025 | 0.109 | 0.23 | 0.820 |
| Household size | -0.013 | 0.021 | -0.59 | 0.553 |
| 1=Native Urdu speaker | -0.008 | 0.103 | -0.07 | 0.942 |
| 1=Tap Water is main source | -0.043 | 0.114 | -0.38 | 0.707 |
| Age in years | -0.006 | 0.007 | -0.84 | -0.020 |
| 1=Female | -0.784 | 0.520 | -1.51 | 0.131 |
| Constant | 1.736 | 0.576 | 3.01 | 0.003 |
| Dependent Var: Treatment (=1 if HH assigned to Treatments (not controls) at baseline) | | | | |

**IV Regression, 1^st^ and 2^nd^ Stage results, predicting Log_10_(*E. coli***) as function of self-reported use, instrumenting self-reports with treatment status.

| **First-stage regressions** |  |  |  |
| --- | --- | --- | --- |
| Number of obs | 2863 |  |  |
| F( 1, 2861) | 186.96 |  |  |
| Prob > F | 0 |  |  |
| R-squared | 0.0613 |  |  |
| Adj R-squared | 0.061 |  |  |
| Root MSE | 0.35018 |  |  |
|  | Coefficient | Std. Err. | t-stat |
| 1=Treatment (vs. Control) | 0.206 | 0.015 | 13.67 |
| Constant | 0.000 | 0.013 | 0 |
| **Instrumental variables (2SLS) regression. Dep var: Log_10_(*E. coli*)** | |  |  |
| Number of obs | 2863 |  |  |
| F( 1, 2861) | 26.19 |  |  |
| Prob > F | 0 |  |  |
| R-squared | 0.1063 |  |  |
| Adj R-squared | 0.106 |  |  |
| Root MSE | 1.3074 |  |  |
|  | Coefficient | Std. Err. | t-stat |
| 1=Self Reports Use in Past 24 Hrs. | -1.397 | 0.273 | -5.12 |
| Constant | 1.286 | 0.049 | 26.4 |
| Instrumented: self-reports treatment in past 24 hours |  |  |  |
| Instruments: 1=Treatment (vs. Control) |  |  |  |
